# Temporary Hospitals

**Published:** 1899-09-09

**Authors:** 


### TEMPORARY HOSPITALS.

The recent prevalence of diphtheria in the district served by the Guildford, Woking, and Godalming Isolation Hospital, has made it necessary for the joint board to consider the question of increased accommodation. At present they are using tents, in addition to the ordinary wards. They have, however, now decided to erect an iron building which is to accommodate 12 beds, six in each ward, with a matron's room. The building is to cost £439, and the foundations about £33, so that here we have a proposal to put up a couple of wards at a total cost of, say, £472, or roughly £40 a bed. This seems a very attractive programme, because poor as corrugated iron may be as a building material in smoky towns, it lasts very fairly in country places. In fact, if durability and cost were all that had to be considered there would be a good deal to be said for cheap so-called temporary hospitals, for the interest of the prime cost of some of our newer permanent hospitals would build a new temporary one every few years, certainly long before the old one was worn out. That, however is not the whole question. In these temporary hospitals which seem so cheap, the cheapness in many cases is, we fear, mainly due to the fact that they do not give the same accommodation as would be looked upon as essential in permanent buildings. If we consider the amount of cubic space which is now given to each case of diphtheria in permanent hospitals, our meaning will be clear enough to anyone who will take the trouble to measure up the capacity of the various tents and iron buildings to which sanitary authorities are so ready to fly because they seem so cheap. If these were to give a couple of thousand cubic feet, with perhaps 12 ft. of wall space to each bed, they also would not be so very cheap.

